# Antibiotic resistant zoonotic bacteria in Irrawaddy squirrel (*Callosciurus pygerythrus*)

**DOI:** 10.1002/vms3.138

**Published:** 2018-11-29

**Authors:** Mohammad Shah Jalal, Md Zohorul Islam, Avijit Dutta, Pangkaj Kumar Dhar, Avijit Das, Mohammad Mahbub Hasan, Himel Barua, Paritosh Kumar Biswas, Abdul Ahad

**Affiliations:** ^1^ Department of Microbiology and Veterinary Public Health Chittagong Veterinary and Animal Sciences University (CVASU) Chittagong Bangladesh; ^2^ Department of Microbiology and Infection Control Statens Serum Institute Copenhagen Denmark; ^3^ Poultry Research and Training Center CVASU Chittagong Bangladesh

**Keywords:** antibiotic resistance, Irrawaddy squirrel, PCR, zoonotic bacteria

## Abstract

Irrawaddy squirrel (*Callosciurus pygerythrus*) may play an important role in the transmission of zoonotic bacteria, but little is known about the carriage of zoonotic bacteria in this common frugivorous rodent in Bangladesh. We aimed to investigate the presence of common zoonotic bacterial pathogens in Irrawaddy squirrel in the southeast part of Bangladesh. A total of 27 rectal and 27 oro‐nasal swabs were collected from 27 healthy wild Irrawaddy squirrels. Four common zoonotic bacteria were isolated following routine laboratory procedures, and were identified based on colony morphology, and biochemical and staining properties. The pathogenic potential of the identified bacteria was confirmed by detection of virulence genes by PCR. All isolates were subjected to antimicrobial susceptibility test against seven antibiotics from six generic groups which are commonly used in human and veterinary medicine in Bangladesh. The prevalence of *Escherichia coli*,* Salmonella* spp., *Yersinia* spp. and *Staphylococcus* spp. was 44.4% (95% CI, 32.0–57.6), 13% (95% CI, 6.1–24.7), 44.4% (95% CI, 32.0–57.6), and 72.2% (95% CI, 59.0–82.5), respectively. We identified potential zoonotic virulence genes in all of these four bacterial species. Antimicrobial susceptibility testing revealed the presence of several multidrug resistant bacterial strains in squirrels. To the best of our knowledge, this is the first report in Bangladesh of the detection of antibiotic resistant zoonotic bacteria in Irrawaddy squirrels. The findings underpin the role of Irrawaddy squirrel as a source of pathogenic antibiotic resistant bacteria, consequently, fruit rejected because of squirrel consumption and squirrel‐bites deserve more concern than previously.

## Introduction

Irrawaddy squirrel (*Callosciurus pygerythrus*), a small‐ to medium‐size rodent in the family *Sciuridae,* is widely distributed in Asian countries including Bangladesh (20°34′N; 92°41′E) (Adhri *et al*. 2015). Squirrels are one of the most common wild rodents that live near human habitants. Recently, they have become a major cause of nuisance in orchards and fruit gardens in Bangladesh; they eat or destroy both green and ripened fruits. Generally, children and many adults directly consume fruits half‐eaten by squirrels. Little is known about the microbial risk of consuming squirrel contaminated half‐eaten fruits. On the other hand, squirrels are also reared as pets in different parts of the world. Furthermore, the ground squirrel animal model is a long‐standing model for the studies of hibernation biochemistry and physiology (Vaughan *et al*. 2006) and it is an emerging model for other medical experimentation (Arendt *et al*. 2003). Thus, squirrel bites are a common health hazard for pet lovers and researchers. However, it is not known whether squirrel carry any antibiotic resistant zoonotic bacteria or not. Therefore, we aimed to investigate the presence of common zoonotic bacteria in Irrawaddy squirrels, and the antibiotic susceptibility of any isolates.

## Materials and methods

### Study design and sample collection

On the basis of a preliminary survey on the abundance of Irrawaddy squirrel, the study was conducted in different environments (e.g. forest, orchards and fruits gardens etc.) of the Cox's bazar (area 1) and the Bandarban district (area 2) of Bangladesh during January 2016 to June, 2017. The live squirrels were captured using steel wire traps (30 cm × 15 cm × 15 cm) and nets, balancing animal welfare and personal safety. Animals were captured in three stages. The first stage was during post‐fruiting period (May, 2016); the second stage was performed in a period of the year when there are relatively fewer fruits available in the study area (October–November, 2016); and the third stage was carried out during fruiting season (April, 2017). The captured squirrels were anaesthetized using cotton wool soaked in Forane (Isoflurane; Abbott Laboratories Ltd.**)** to avoid any hazard related to squirrel bites. One rectal swab and one oro‐nasal swab samples were collected using sterile cotton tipped swab stick in sterile 50 mL Falcon Tubes containing 10 mL Buffered Peptone Water (Oxoid Ltd., P^H^: 6.2 ± 0.0) and/or in 1.5 mL Eppendorf tube containing 1 mL Stuart's Transport Media. Samples were immediately transported to the laboratory of Department of Microbiology and Veterinary Public Health (DMVPH), Chittagong Veterinary and Animal Sciences University (CVASU), Chittagong, Bangladesh, for microbiological analysis. After sample collection, the squirrels were fed and kept in a comfortable cage until being able to normally move, and then they were released within their natural habitat.

### Isolation and identification of bacteria

Four selective bacteria (*Escherichia coli, Salmonella* spp.*, Yersinia* spp. and *Staphylococcus* spp.) were isolated from swab samples, following standard laboratory protocols. The mentioned bacteria were identified based on colony morphology on selective media, biochemical and staining properties (Carter *et al*. 1995), and Polymerase Chain Reaction (PCR) of species‐specific pathogenic genes (Table 1). Identified bacterial isolates were preserved at −80°C in brain heart infusion broth with 50% glycerine for further use.

**Table 1:** Sequences of primers used to detect species‐specific pathogenic gene(s) in *Escherichia coli*,* Salmonella* spp., *Yersinia* spp. and *Staphylococcus* spp

Organism	Target Gene	Primer Sequence	Annealing Temp (°C)	Amplicon (bp)	Reference
*E. coli*	*hly*	**F:** ACGATGTGGTTTATTCTG GA	58	165	DesRosiers *et al*. 2001;
		**R:**CTTCACGTGACCATACAT AT			
	*stx1*	**F:**ACACTG GATGATCTCAGTGG	58	614	DesRosiers *et al*. 2001;
		**R:**CTGAATCCCCCTCCATTATG			
	*stx2*	**F:**CCATGACAACGGACAGCAGTT	58	779	Islam *et al*. 2015;
		**R:**CCTGTCAACTGAGCAGCACTTT			
	*eae*	**F:**CCCGAATTCGGCACAAGCAT	59	881	Oswald 2000;
		**R:**CCCGGATCCGTCTCGCCAGTA			
*Salmonella* spp.	–	**ST11:**AGCCAACCATTGCTAAATTGGCGCA	60	429	Aabo *et al*. 1993;
		**ST15:**GGTAGAAATTCCCAGCGGGTACTG			
*Yersinia* spp.	*pla*	**F:**ATCTTACTTTCCGTGAGAAG	55	478	Hinnebusch & Schwan 1993;
		**R:**CTTGGATGTTGAGCTTCCTA			
	*ail*	**F:**TAATGTGTACGCTGCGAG	57	351	Thoerner *et al*. 2003;
		**R:**GACGTCTTACTTGCACTG			
*Staphylococcus* spp.	*fnb*A	**F:**GCGGAGATCAAAGACAA	50	1279	Signas *et al*. 1989
		**R:**CCATCTATAGCTGTGTGG			

Temp, temperature; °C, degree centigrade; bp, base pair; F, Forward primer; R, Reverse primer.

### Polymerase chain reaction (PCR)

Bacterial colonies grown overnight on blood agar plates were used for DNA extraction by boiling lysates method, according to previously described protocol (Islam *et al*. 2015). PCR was performed to detect species specific pathogenic gene(s) in bacteria using previously reported standard primer sequences and protocols (Table 1). In brief, 25 *μ*L PCR mix was prepared by mixing 12.5 *μ*L ready to use master mix (Thermo Scientific Ltd., USA), 9.5 *μ*L nuclease‐free water, 1 *μ*L of each primer, and 1 *μ*L DNA template. PCR amplification was performed on a thermo‐cycler (Applied Biosystem, 2720 thermal cycler, Singapore) using 94–95°C denaturation temperature, 50–60°C annealing temperature and 72°C extension temperature for 30–35 cycles.

### Antimicrobial susceptibility test

All cultured isolates were tested for antimicrobial susceptibility profile using a panel of seven antibiotics within six generic groups by standard disc diffusion according to Kirby‐Bauer method (Bauer *et al*. 1966). Zone diameters were measured and interpreted following Clinical Laboratory and Standards Institute guideline (CLSI, 2016).

### Data analysis

All attributed data were recorded in an Excel spread sheet (Excel 2007, Microsoft Corporation, USA) and subjected to statistical analysis. Logistic regression and Pearson's Chi‐squared (two tailed) test was performed in R (version 3.3.2). The 95% confidence interval of the prevalence values were calculated by the modified Wald method using QuickCalcs in the Graph Pad software.

## Results

### Overall prevalence

We collected 54 swabs (27 rectal, 27 oro‐nasal) from 27 squirrels. *E. coli*,* Salmonella* spp., *Yersinia* spp. and *Staphylococcus* spp. were isolated from 24 (3 oro‐nasal, 21 rectal), 7 (0 oro‐nasal, 7 rectal), 24 (9 oro‐nasal, 15 rectal) and 39 (19 oro‐nasal, 20 rectal) samples, respectively. A comprehensive result of bacteriological analysis is shown in Table 2. The overall prevalence of these four bacteria was relatively higher in rectal swab samples (58.3%) compared to oro‐nasal swabs (28.7%). The prevalence of *E. coli*,* Salmonella* spp., *Yersinia* spp. and *Staphylococcus* spp. in oro‐nasal swabs were 11.1%, 0%, 33.3% and 74.1%, respectively. Conversely, the prevalence of *E. coli*,* Salmonella* spp., *Yersinia* spp. and *Staphylococcus* spp. in rectal swabs were 77.8%, 25.9%, 55.6% and 74.1% respectively. Although, the presence of *E. coli* and *Salmonella* spp. was significantly (*P* < 0.05) related to type of sample, the presence of *Yersinia* spp. and *Staphylococcus* spp. was not related to this variable. The prevalence of *Yersinia* spp. and *Staphylococcus* spp. in Irrawaddy squirrel was significantly (*P* < 0.05) related to the season in the study area, while the isolation of *E. coli* and *Salmonella* spp. showed no significant relationship with seasons. There was no significant effect of sex on the prevalence of any of the four species of bacteria, although male squirrels had relatively higher loads of *Staphylococcus* spp., but with a borderline significance (*P* = 0.083).

**Table 2:** Relationship between different categorical variables and prevalence of bacteria identified, based on colony morphology, staining and biochemical properties

Variables	Categories	*N*	Proportionate Prevalence							
			*E. coli*		*Salmonella* spp.		*Yersinia* spp.		*Staphylococcus* spp.	
			PP (95% CI)	*P*	PP (95% CI)	*P*	PP (95% CI)	*P*	PP (95% CI)	*P*
Season	May, 2016	8	37.5 (13.5–69.6)	0.533	0.0 (0.0–37.2)	0.352	0.0 (0.0–37.2)	0.016*	12.5 (0.1–49.2)	0.000*
	Oct–Nov, 2016	14	57.1 (32.6–78.7)		21.4 (6.8–48.3)		42.9 (21.3–67.5)		50.0 (26.8–73.2)	
	April, 2017	32	40.6 (25.5–57.8)		12.5 (4.4–28.7)		56.2 (39.3–71.9)		100 (87.3–100)	
Sex	M	30	43.3 (27.4–60.8)	0.854	13.3 (4.7–30.4)	0.928	46.7 (30.2–63.9)	0.713	83.3 (66.0–93.1)	0.083
	F	24	45.8 (27.9–64.9)		12.5 (3.5–31.8)		41.7 (24.4–61.2)		62.5 (42.6–78.9)	
Sample	ON	27	11.1 (3.0–28.9)	0.000*	0.0 (0.0–14.8)	0.005*	33.3 (18.5–52.3)	0.100	74.1 (55.1–87.1)	1.000
	R	27	77.8 (58.9–89.7)		25.9 (12.9–44.9)		55.6 (37.3–72.4)		74.1 (55.1–87.1)	
Total		54	44.4 (32.0–57.6)		13.0 (6.1–24.7)		44.4 (32.0–57.6)		72.2 (59.0–82.5)	

*, significant *P*‐value; ON, Oro‐nasal swab; R, rectal swab; M, male squirrel; F, female squirrel; %, percentage; *N*, number of samples; PP, prevalence; *P*, probability value; CI, confidence interval.

### Detection of virulence genes

Among 24 culture and biochemically positive *E. coli* isolates, eight isolates (33.3%; 95% CI, 17.8–53.4) carried shiga toxin producing gene (*stx2*); one (4.2%; 95% CI, 0.01–21.8) isolate carried the intimin encoding gene (*eae*). Furthermore, one isolate harboured both *stx2* and *eae* genes. Two (28.6%; 95% CI, 7.6–64.8) of seven culture‐positive *Salmonella* spp. were positive for *ST* DNA fragment. One (4.2%; 95% CI, 0.01–21.8) of 24 tentatively identified *Yersinia* spp. was carrying the plasminogen activator protein (*pla*) encoding gene while another two (8.3%; 95% CI, 1.2–27.0) were harbouring the attachment invasion locus protein (*ail*) producing gene. Although *Staphylococcus* spp. was the most prevalent bacteria isolated from squirrel, only one (2.6%; 95% CI, 0.01–20.6) of 39 culture‐positive isolates was positive for the fibrinogen binding protein A (*fnb*A) encoding gene (Table 3).

**Table 3:** Presence of virulence genes in isolated bacteria

Organism	*N*	Target genes	Positive samples	Proportion	95% CI
*E. coli*	24	*hly*	0	0.0	0.0–16.3
		*stx1*	0	0.0	0.0–16.3
		*stx2*	8	33.3	17.8–53.4
		*eae*	1†	4.2	0.01–21.8
*Salmonella* spp.	7	*ST* *	2	28.6	7.6–64.8
*Yersinia* spp.	24	*pla*	1	4.2	0.01–21.8
		*ail*	2	8.3	1.2–27.0
*Staphylococcus* spp.	39	*fnb*A	1	2.6	0.01–20.6

*N*, No. of culturally and biochemically positive samples; CI, Confidence interval.

*ST is a genus‐specific DNA fragment of *Salmonella*.

One sample carried both *eae* and *stx2* genes.

### Antimicrobial resistance pattern of isolated bacteria

**Figure 1:**
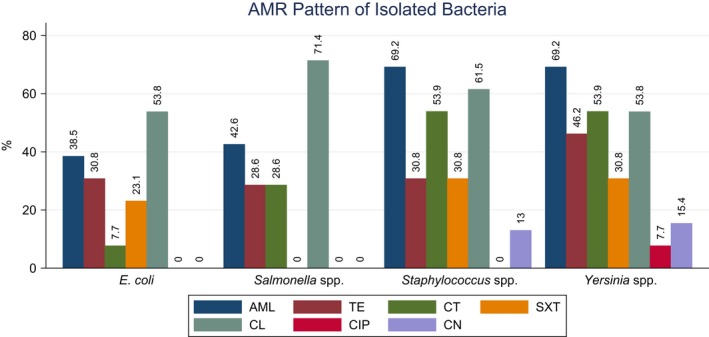
Antibiotic resistance pattern (expressed in percentage) of *Escherichia coli*,* Salmonella* spp., *Staphylococcus* spp. and *Yersinia* spp., isolated from Irrawaddy Squirrel, against AML, amoxicillin; CIP, ciprofloxacin; CL, cephalexin; CN, gentamycin; CT, colistin sulphate; SXT, sulphamethoxazole‐ trimethoprim; TE, tetracycline.

All culturally positive bacterial isolates were tested for antimicrobial susceptibility for a panel of seven commonly used antibiotics under six generic groups. Test results are represented in Fig. 1. During this study, *E. coli* showed highest resistance to colistin sulphate (53.8%) and sensitive to gentamicin (100%). About 71.4% *Salmonella* spp. were resistant to colistin sulphate while most of the *Salmonella* isolates were sensitive to gentamycin (100%), sulphamethoxazole‐ trimethoprim (85.7%). *Yersinia* isolates were resistant to amoxicillin (69.2%) but sensitive to gentamicin (84.6%). *Staphylococcus* spp. isolates were most likely sensitive to ciprofloxacin (92.3%). Bivariate logistic regression analysis revealed that *Yersinia* spp. and *Staphylococcus* spp. were relatively more likely to be resistant to the antibiotics tested, but *Salmonella* spp. isolates also showed almost similar resistance pattern of *E. coli* (Table 4)

**Table 4:** Bivariate relationship between different categorical variables and AMR of bacteria. *E*. *coli*, rectal swab and area‐1 were used as reference value for organisms, sample type and location, respectively, to determine the association of these categorical variables with the resistance of bacteria to different antibiotics

Categories	Antimicrobials													
	SXT		CT		AML		TE		CIP		CL		CN	
	OR (95% CI)	*P*	OR (95% CI)	*P*	OR (95% CI)	*P*	OR (95% CI)	*P*	OR (95% CI)	*P*	OR (95% CI)	*P*	OR (95% CI)	*P*
(A) Organisms														
*E*. *coli*	Ref		Ref		Ref		Ref		Ref		Ref		Ref	
*Salmonella* spp.	0.6 (0.0–6.6)	0.642	4.8 (0.4–65.8)	0.240	2.1 (0.3–15.3)	0.448	0.5 (0.1–3.3)	0.448	4.8 (0.3–65.8)	0.240	1.0	NA	1.0	NA
*Yersinia* spp.	1.4 (0.3–8.5)	0.659	14 (1.4–141.5)	0.025*	4.7 (0.7–30.3)	0.102	1.4 (0.3–6.4)	0.695	5.3 (0.5–56.2)	0.164	0.1 (0.0–1.4)	0.090	0.3 (0.0–1.9)	0.197
*Staphylococcus* spp.	2.1 (0.4–11.5)	0.399	14 (1.4–141.5)	0.025*	1.9 (0.4–9.6)	0.423	0.7 (0.1–3.5)	0.692	1.0 (0.1–17.9)	1.000	0.5 (0.0–5.8)	0.547	1.0	NA
(B) Sample type														
R	Ref		Ref		Ref		Ref		Ref		Ref		Ref	
ON	0.5 (0.1–2.0)	0.302	1.6 (0.4–5.4)	0.487	0.4 (0.1–1.4)	0.157	2.2 (0.6–7.6)	0.206	4.1 (0.8–20.1)	0.083	0.2 (0.0–1.3)	0.083	3 (0.6–15.5)	0.190
(C) Location														
Area 1	Ref		Ref		Ref		Ref		Ref		Ref		Ref	
Area 2	1.0 (0.3–4.1)	0.975	0.9 (0.3–3.4)	0.908	1.9 (0.4–8.4)	0.384	0.4 (0.1–1.5)	0.184	0.3 (0.0–2.5)	0.250	0.7 (0.1–3.3)	0.634	0.3 (0.0–3.1)	0.332

*, Significant *P*‐value; ON, oro‐nasal swab; OR, odd ratio; NA, not applicable; R, rectal swab; *P*, probability value; Ref, reference value.

### Multi‐drug resistance (MDR) of isolated bacteria

The drug resistance pattern of isolated bacteria shows that the Irrawaddy squirrel can carry multi‐drug resistant bacteria. From 27 Irrawaddy squirrel, four *E. coli*, four *Salmonella* spp., six *Yersinia* spp. and six *Staphylococcus* spp. were isolated that showed resistance against three or more unrelated groups of antibiotics (Table S1).

## Discussion

The result of this study revealed that Irrawaddy squirrels can carry *Escherichia coli*,* Salmonella* spp., *Yersinia* spp. and *Staphylococcus* spp. Bacterial load was higher in rectal swabs (58.3%) in comparison to oral swabs (28.7%). *E. coli* is the predominant facultative anaerobic organism among the aerobic commensal flora in gut of human and animal (Chart 2002; Russo & Johnson 2003).

Among all strains, a group of *E. coli,* the Shiga toxin producing *E. coli* (STEC), have recently received particular attention due to the serious clinical implications associated with large scale epidemics in humans attributed to this pathogen. Along with many other virulence markers such as enterohemolysin, catalase‐peroxidase, adhesion, intimin etc. (Etcheverria & Padola 2013), STEC produces two major groups of Shiga toxins, Stxl and Stx2 (Kuntz & Kuntz 1999) which are responsible for severe diseases, such as haemolytic uremic syndrome, in humans (Griffin & Tauxe 1991; Friedrich *et al*. 2002). In this study, the presence of *stx2* gene in eight (33.3%, 95% CI, 17.8–53.4) *E. coli* isolates was confirmed by PCR; which indicates the potential significance of squirrels carrying the virulent strain as the strain producing Stx2 alone is considered more virulent than those producing Stx1 alone, or Stx1 and Stx2 in combination (Donohue‐Rolfe *et al*. 2000). Furthermore, the presence of the intimin encoding gene (*eae* gene) in the same isolate enhanced the probability of the existence of virulent and zoonotic strains of *E. coli* in squirrels. However, there was no isolate showing positivity for haemolytic properties on blood agar or *hly* and/or *Stx1* gene(s) in PCR. This suggests that this wild species may not carry *E. coli* O157 strain. *E. coli* isolated from squirrels showed resistance to many commonly used antibiotics in human and veterinary medicine such as‐ colistin sulphate (53.8%), amoxicillin (38.5%) and tetracycline (30.8%).

Antimicrobial resistance (AMR) was relatively, but not significantly, higher in squirrels captured from area 1, which has previous history of poultry farms in the area. Three out of four multi‐drug resistant *E. coli* were isolated from samples collected from area 1. So, it can be hypothesized that squirrels acquired their antibiotic‐resistant bacteria from environmental sources contaminated by drug resistant *E. coli* strain from poultry farms, although further research needs to be done.

Water‐borne and enteric bacteria *Salmonella* spp. can cause diseases in humans, mammals and poultry. It is an important zoonotic bacteria and in this study, seven (25.9%; 95% CI, 12.9–44.9) rectal swab samples were found culture‐positive for *Salmonella* spp. Among them, the presence of genus‐specific DNA portion ST (*ST11* and *ST15*) in two samples confirmed the isolates as pathogenic *Salmonella* spp. (Aabo *et al*. 1993). However, a further study is required to identify them fully. *Salmonella* spp.*,* also, were resistant to colistin sulphate (71.4%) but sensitive to gentamicin (100%). The almost similar antimicrobial resistance of both *E. coli* and *Salmonella* spp. isolates may be due to their phylogenetic relatedness and/or their emergence from similar source. It may be a consequence of inter‐species resistance gene transfer although this needs to be proved by further research.

*Yersinia* is a genus of important and zoonotic significant bacteria that includes *Y. pestis, Y. enterocolitica* and *Y. pseudotuberculosis*. In this study, 24 (9 oro‐nasal, 15 rectal) samples were found to be culture positive for *Yersinia* spp. One isolate (4.2%; 95% CI, 0.01–21.8) was confirmed as *Y. pestis* through detection of *pla* gene with an amplicon size 478 bp. *pla* gene encoding plasminogen activating proteins (coagulase and fibrinolysin) which is found in pathogenic strains of *Y. pestis* (Kukkonen *et al*. 2001). However, recent findings suggest that *pla* gene is not specific to *Y. pestis* as it is also found in *E. coli* and *Citrobacter koseri* (Hansch *et al*. 2015). The *ail* gene encodes Attachment invasion locus protein (an outer membrane protein that helps bacteria to invade host cells) and is found in pathogenic strains of *Y. enterocolitica* (Sihvonen *et al*. 2011). In this study, two (8.3%; 95% CI, 1.2–27.0) isolates were confirmed as *Y. enterocolitica* as they were positive for *ail* gene in PCR assay (Miller *et al*. 1989). So, the present findings indicate that Irrawaddy squirrel can carry pathogenic and zoonotic *Yersinia* bacteria. However, we found no isolate that fully coincided with the cultural and biochemical properties of *Y. pseudotuberculosis*. *Yersinia* spp. showed the highest resistance to amoxicillin (69.2%) and highest sensitivity to gentamicin (84.6%). This may be due to the long‐term use of *β*‐lactam antibiotics in human and veterinary medicine, through gene transfer or mutation or selection pressure, and spread of amoxicillin‐resistant *Yersinia* spp. within the squirrel population. *Staphylococcus* spp. is one of the most prevalent bacteria isolated from animals (including squirrel) and human bite wounds (Goldstein *et al*. 1978). During this study, *Staphylococcus* spp. was isolated from 39 (72.2%) samples. Presence of *fnb*A gene in one sample (2.6%; 95% CI, 0.01–20.6) is an indication of pathogenic Staphylococcal carriage by Irrawaddy squirrels. The isolated staphylococci in this study were mostly resistant to amoxicillin (69.2%) and sensitive to ciprofloxacin (92.3%). This may reflect contamination of the natural environment with bacteria that have already acquired resistance against *β*‐lactam antibiotics as a consequence of their long term and widespread use in human and veterinary medicine.

During this study, multi‐drug resistance (MDR) was observed in all four types of bacteria. In present study, four (16.7%; 95% CI, 6.1–36.5) *E. coli* isolates, three (42.9%; 95% CI, 15.8–75.0) *Salmonella* spp. isolates, six (25%; 95% CI, 11.7–45.2) *Yersinia* spp. isolates and six (15.4%; 95% CI, 6.9–30.1) *Staphylococcus* spp. isolates were found resistant to three or more unrelated groups of antibiotics at a time, *in vitro*. This finding supports the existence of MDR in wild‐caught squirrels (Cloud‐Hansen *et al*. 2007), that challenges the treatment of any illness resulting from the consumption of squirrel rejected half‐eaten fruits or squirrel dung contaminated water or from squirrel bites during handling, in both humans and livestock. The bacterial load in squirrel was significantly related to the seasons. According to findings of this study, more bacteria were isolated during the fruiting period (April) compared to post‐fruiting (May) or off fruiting (October to November) periods. So, it can be assumed that there is a probability of transmission of pathogenic bacteria from squirrels to humans, if people eat squirrel rejected or half‐eaten fruits.

## Conclusion

Irrawaddy squirrel can carry, in oral or faecal material, pathogenic and zoonotic bacteria such as *E. coli, Salmonella* spp., *Yersinia pestis, Y. enterocolitica, Staphylococcus aureus* etc. As all these bacteria may be associated with illness in humans and domestic animals, it is important to be concerned about the consumption of squirrel rejected fruits and squirrel bite wound management. The bacteria may acquire transposon‐mediated resistance genes from environmental bacteria which have already acquired resistance from human or animal pathogens and/or from genetic mutations. Although bacteria carried by Irrawaddy squirrel can be resistant to many commonly used antibiotics, gentamicin or ciprofloxacin are, at present, relatively effective against carried bacteria.

## Source of funding

Advanced Studies and Research, Chittagong Veterinary and Animal Sciences University.

## Conflicts of interest

None.

## Contribution

MSJ designed and carried out this project. ADu, PKD, ADa, MMH contributed in samples collection, data collection and lab works, in a team. MSJ and MZI analyzed data and wrote this article; and HB, PKB, AH supervised the project and checked the writing, of this article.

## Ethics statement

We captured live squirrels from the selected study areas of Bangladesh with a formal permission from Bangladesh Forest Department, and Samples were collected whilst assuring animal welfare. This project was approved by the ethical committee of Chittagong Veterinary and Animal Sciences University, Bangladesh.

## Supporting information


**Table S1.** Multi‐drug resistant pattern of isolated bacteria
