# Supplementary material for: Antibiotic resistant zoonotic bacteria in Irrawaddy squirrel (Callosciurus pygerythrus)
Source: Vet Med Sci. 2018 Nov 29;5(2):260–8. doi: 10.1002/vms3.138 (PMC6556763; doi:10.1002/vms3.138)
Supplement: Supplementary file 1 — Table S1. Multi‐drug resistant pattern of isolated bacteria [file VMS3-5-260-s001.doc]

**Supplementary Table:** Multi-drug resistant pattern of isolated bacteria

| **Bacteria** | **Sample** | **%(95% CI)** | **Resistant Antibiotics** | **No. Groups of Antibiotics** |
| --- | --- | --- | --- | --- |
| *E. coli* | EF10 |  | SXT,AML,TE,CL | 3 |
|  | EO9 | 16.7(6.1-36.5) | CT,AML,TE,CIP,CL | 4 |
|  | EF16 |  | SXT,TE,CL | 3 |
|  | EF14 |  | SXT, AML, TE | 3 |
| *Salmonella* spp | SF9 |  | SXT,CT,AML,CL | 3 |
|  | SF12 | 42.9(15.8-75.0) | CT,AML,CIP,CL | 3 |
|  | SF13 |  | TE,CIP,CL | 3 |
| *Yersinia* spp | Y1 |  | SXT,CT,AML,TE,CIP,CL | 5 |
|  | Y12 |  | CN,AML,TE,CL | 3 |
|  | Y20 | 25(11.7-45.2) | CT,AML,TE,CL | 3 |
|  | Y11 |  | SXT,CN,CT,AML,TE | 4 |
|  | Y15 |  | SXT,CT,AML,CIP,CL | 4 |
|  | Y32 |  | SXT,CT,AML,CL | 3 |
| *Staphylococcus* spp | STO19 |  | SXT,CN,CT,TE,CL | 5 |
|  | STO12 |  | SXT,CN,CT,AML,TE,CL | 5 |
|  | STO7 | 15.4(6.9-30.1) | CN,CT,AML,TE,CL | 4 |
|  | STO21 |  | CN,CT,CIP,CL | 4 |
|  | STO26 |  | SXT,CT,AML,CL | 3 |
|  | STO10 |  | SXT,CN,TE,CL | 4 |

AML=Amoxicillin; CL=Cephalexin; TE=Tetracycline; CT= Colistin sulfate; CN=Gentamycin; CIP= Ciprofloxacin; SXT=Sulphamethoxazole- trimethoprim; %=Percentage; CI=Confidence interval.
